# Medical Miscellany

**Published:** 1826-07

**Authors:** 


### XIII.

### MEDICAL MISCELLANY.

### ENGLISH MEDICAL ETYMOLOGIES.

A. *Arabic:* Arm. *Armoric:* B. *Belgic:* Copt. *Coptic:* D. *Danish:* F. *French:* G. *Gothic:* Hind. *Hindoostanee:* Isl. *Islandic:* It. *Italian:* L. *Latin:* L. B. *Barbarous Latin:* M. G. *Mæso-Gothic:* O. E. *Old English:* P. *Persian:* Sans. *Sanscrit:* S. *Saxon:* Scot. *Scottish:* Sp. *Spanish:* Swed. *Swedish:* T. *Teutonic:* W. *Welch.*

AGUE, an intermittent fever, accompanied with cold and hot fits; L. *acuta febris* is supposed to have produced our word, but the disease has never been considered as acute. The vulgar call it *agur*, and Isl. *agur*, *ogur:* S. *ega*, *oga*, terror, tremor, may have had *hri*, *hrath*, an attack, a fit, added to it, corresponding with F. *acce*, which is the *accessus* of Pliny.

ALCHYMY, the highest chemistry: A. *al keemiya*, from A. and Copt. *hem*, *khem*, heat, process of heat, *al khum*, an alembic: but some suppose that the A. article has been prefixed to χεῦμα.

BODY, the human stature or person; S. *bodig;* T. *potih*, signified the human person or stature only; and there does not appear to be any cognate term, unless *bode* might have been considered as the residence of life, which in all G. dialects is synonymous with what we call body. P. *body;* Sans. *bodaun*, a being, may possibly have a common origin with our verbs to *be* and to *bide*.

BONE, the most solid part of an animal's body: G. *bein*, *bun;* Swed. *ben;* B. *been;* S. *ban;* T. *bein;* all of which, like L. *tibia*, signify a leg and pipe or tube.

BRAIN, the soft substance within the scull; G. *huarn;* S. *hierna;* O. E. *harne;* B. *brein;* S. *bragin;* T. *pregin*, corresponding with Περικράνιον.

CHILD, an infant; G. *kyld, kulld*, from *eld*, a fœtus; S. *cild;* G. *kylla*, to beget. *Kyld* signified particularly legitimate offspring; but *bairn* was applied to any condition; Scot. *chiel*, Sp. *chula*, a youth.

CHINCOUGH, a convulsive cough; T. *kink host;* Swed. *kikhosta;* F. *quinte;* Scot. *kink* is applied to a violent fit of coughing or laughing, from G. *kaugian, kikna;* B. *kichen;* S. *aceocan*, to kill by stopping the breath.

COUGH, a convulsion of the lungs; A. *qubhu;* Hind. *kuf;* G. *kuef;* B. *kuch;* the G. word signifies suffocation.

GALL, bile: Isl. *gall;* Swed. *galla;* D. *gald;* S. *gealla;* B. *gal;* χολή; S. *geal*, is yellow.

GOUT, arthritis, a periodical disease attended with great pain; F. *goutte;* It. and Sp. *gotta, gota*, from L. *gutta*, on the supposition that it was occasioned by the distillation of catarrhal humours in the joints; Swed. *gickt;* T. *gicht;* S. *gechta;* are also names for the gout; but, like B. *jeukt;* Scot. *youk*, they signify itching.

HICCUP, a convulsion of the stomach, the yex; P. *hukkak;* Swed. *hicka;* G. *hixt;* T. *hix;* B. *hik, hickse;* Arm. *hic;* W. *ig;* L. B. *hoqueta;* F. *hoquet;* Yex is from G. *hex, hixt;* D. *hikkes;* B. *hickse;* S. *geocsa.*

ITCH, a cutaneous disease, attended with a desire of scratching; G. *ikt;* Isl. *gicht;* Swed. *gickt;* S. *gichta;* B. *jiochte;* T. *jucke;* Scot. *yuke*. It denoted with the Goths any disorder of the skin, including leprosy, and afterwards also the gout; but the origin of our word is apparently from *eat*, in the same way that we use *mange* from F. *manger*, to eat, to itch.

JAUNDICE, a distemper of the liver, accompanied by a yellowness of the skin; F. *jaunisse; jaune, jaulne*, yellow; from G. *gul;* S. *geolew;* It. *giallo.*

JULAP, a liquid form of medicine; P. *gulab*, from *gul*, a rose, and *ab*, water; L. B. *julapium.*

KIDNEY, a gland in the reins; G. *kuid*, the belly, and *nare;* D. *nyre;* T. *nier;* S. *nœr*, the reins.

LEECH, a medical man, a surgeon; M. G. *lek, leikeis;* T. *leck;* S. *læc, lece;* G. and Swed. *lækare, læknare;* Sclav. *likœr*, apparently from G. *laka*, to diminish, and called in L. B. *minutor*, a blood-letter; S. *lecan*, to diminish or make small; *læcefinger*, the little finger. The animal, in S. *læce, lyce*, is so named from its use in benefiting health, by drawing blood.

LUNGS, the organs of respiration; Swed. *lunga;* D. and T. *lunge;* S. *lungen;* B. *long;* G. *lugu*, the air.

MEASLES, an eruptive disease; T. *masel;* B. *mazelen;* D. *meisling;* Arm. *mezell;* F. *mezel*, leprosy. T. *mase;* L. *maculosus*, signify spotted; but G. *missli, mislitur*, discolour, may be the etymon.

MOUTH, the aperture in the head where food is received; Sans. *moonh*; Hind. *munh*; G. *mun*, *munth*; Swed. *mun*; T. *mund*; B. *mond*; M. G. *munths*; S. *muth*; Arm. *muzz*; Scot. *mow*; F. *moue*. The word appears to originate from G. *in*, int, an entrance; whence G. *minn*; Swed. *mynne*, *myning*, an orifice, an opening inward; and G. *mund*, *mynd*, like L. *os*, signified the countenance.

We have made these selections from a very remarkable work, recently published, which we might designate a System of English Etymology,* in which the true principles of etymological investigation are unfolded, with a simplicity of description, a precision of application, and a comprehensiveness of research, in all respects extraordinary: it is difficult to say, indeed, which is most conspicuous in this volume, the author's great talents, his vast erudition, or the purity of his philosophical deductions: the book itself, we are sure, is altogether original, elaborate, excellent.

---

VIPER-BITE—SUCCESSFUL APPLICATION OF THE CUPPING-GLASS.

At a meeting of the Academy of Medicine at Paris on the 25th May, Mons. Piorry presented to that learned body a man who had been bitten by a *viper* on the 17th of the same month, and who still, as it would seem, was suffering under some of the effects of that accident.

The bite had, it appears, been inflicted about half past five P. M.; and, at *eight*, when the patient was seen by M. Piorry and two other physicians, his situation was such as to create the strongest apprehensions for his safety. A *cupping-glass*, therefore, was immediately applied to the wound, according to the plan lately proposed and experimentally acted upon in Paris by Dr. Barry, and with the happiest effects.

The relief, however, thus procured, was not, it appears, altogether compleat or permanent, in consequence, probably, of the length of time which had elapsed before the application of the cupping-glass, and the want of practical skill, perhaps, and knowledge on the part of the operators, to whom the proceeding was, of course, entirely new.†

Some unpleasant symptoms, therefore, arose which rendered the repeated application of leeches necessary and retarded the cure, but the man, when exhibited at the academy, was manifestly no longer in any kind of danger.

---

* Etymons of English Words; by the late John Thompson, M. R. I. and A. S. Private Secretary to the Marquis of Hastings in India: 4to. London and Edinburgh, 1826. Mr. T. was distinguished by the highest characters of a gentleman and a scholar: his work was left in MS. at his death, in a state fit for the press, and has since been published by his family, in a manner exceedingly creditable to their learning and filial piety.

† Globe de Paris, Mai 23.

### ANGINA PECTORIS.

Dr. Bruckmann of Brunswick has made some observations on angina pectoris in the Journal Complémentaire for April 1826, which we shall just glance at here.

He says, what is indeed true enough, that writers have differed very widely on the cause of this complaint; but we would beg to ask on what subject in medicine have writers not differed? Dr. B. farther observes that in his experience, though the greater number of patients have died from this affection, yet many have recovered. As an instance of recovery he adduces his own case. It is this: The first attack our author experienced was on ascending a considerable height on a cold March morning with the wind to the eastward. Since that time he was more or less subject to paroxysms, particularly in the night; these would come on with an intolerable sense of pressure on the sternum, anxiety, sinking, a kind of paralytic feeling in the arms, and the pulse small and spasmodic, beating from 65 to 80 in the minute. In a quarter or half hour, all this would go off, and sleep return. Since 1824 these attacks have disappeared. Dr. B. remarks that, previously to this complaint, he had been subject to rheumatism, sciatica, intermissions and extreme slowness of the pulse and catarrhal affections.

That this was not an instance of the affection in question, as depending on organic disease, we think there can be little doubt. In all probability this was but one form of that Proteian malady, INDIGESTION. It might have been of a rheumatic origin, for Dr. B. was, it will be observed, very subject to rheumatism; at all events we are sure that no organic disease was present.

But the fact is, that every symptom which appears in fatal angina pectoris, dependent on some lesion of structure in the heart and great vessels, will be generated by irritation in the stomach. A physician of this metropolis had the most exquisitely marked symptoms of this terrible disease, so much so that some of the first medical characters advised him to leave England and go to the South of France, there to live as long, and die as easy, as he could. This being their opinion he left off medicine and restrained himself to the most rigid regimen, leaving off all wine or other stimulants. The consequence was, a complete recovery.

---

### TRANSFUSION.

An ingenious apparatus for performing this operation has been lately produced by Mr. Scott, a surgeon of London, which consists of a tubulated funnel (supported on a metallic stand) to receive and convey the blood into the lower chamber of a pump by which it is propelled, through a short flexible tube, into a vein of the patient. This tube is armed with a silver pipe, tapering to a point with an oblique oval aperture at each side, and curved at an obtuse angle, ¾ of an inch below the extremity: behind this curve, a broad shield intersects the pipe in a diagonal direction. This structure not only enables the surgeon to pass the pipe with facility through the opening made by a lancet in the vein (without laying it bare) but allows it to be lodged securely within it, the current of blood being directed by the oblique position of the apertures, directly forwards in the course of the vein; whilst its regurgitation is prevented by the pressure of the shield upon the external opening. There is also another armed flexible tube for taking the blood, if preferable, immediately from the vein without atmospheric communication.

This apparatus has been attached so correctly by Mr. Read, to his patent syringe, that the whole is mathematically true, and blood may be transmitted from one person to another without the injection of a globule of air, a circumstance essentially necessary in an operation of this nature.

